# Hearing Improved

**Published:** 1857-12

**Authors:** 


### HEARING IMPROVED.

Tie a thread to a ball of cotton wool as large as a pea; put the other end of the thread through a small silver or other tube, then draw it up, until the cotton touches the end of the tube; next wet the cotton with glycerine, then introduce the tube carefully in the ear with the cotton foremost, and when, by the improved hearing, it is ascertained that the cotton is in its right place—that is against the "drum of the ear"—withdraw the tube, the thread remaining, hanging out of the ear an inch or more, for withdrawing the cotton when it gets dry.

Tepid water has been used by European physicians to wet the cotton; but we suggest the employment of glycerine for that purpose, as it is clear as water, in every way harmless, and remains moist longer than any liquid known which is, at the same time, so entirely innocuous.

Returning imperfection of hearing will indicate the drying of the cotton, and the necessity of replacing it.

A London surgeon obtained great notoriety several years ago by his success in removing comparative deafness of long continuance, by introducing a few drops of glycerine into the ear every day: the philosophy of it was, its long moisture powers not only dissolved the hardened wax, but counteracted that heat and dryness of the parts which caused the wax of the ear to harden as soon as it was secreted.

This use of glycerine, which is nothing more than the sweet principle of oils, in connection with the cotton, tube, and thread, is a suggestion of our own, and we throw it out as a hint for professional experiment.

